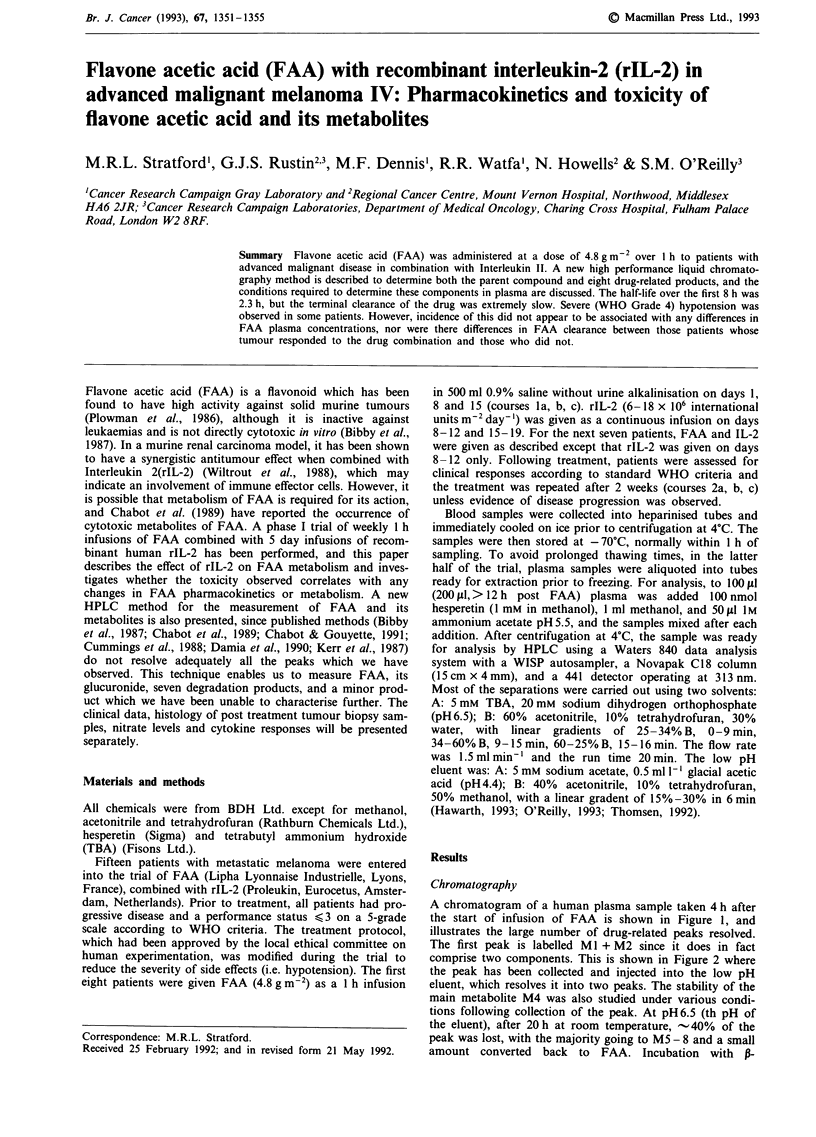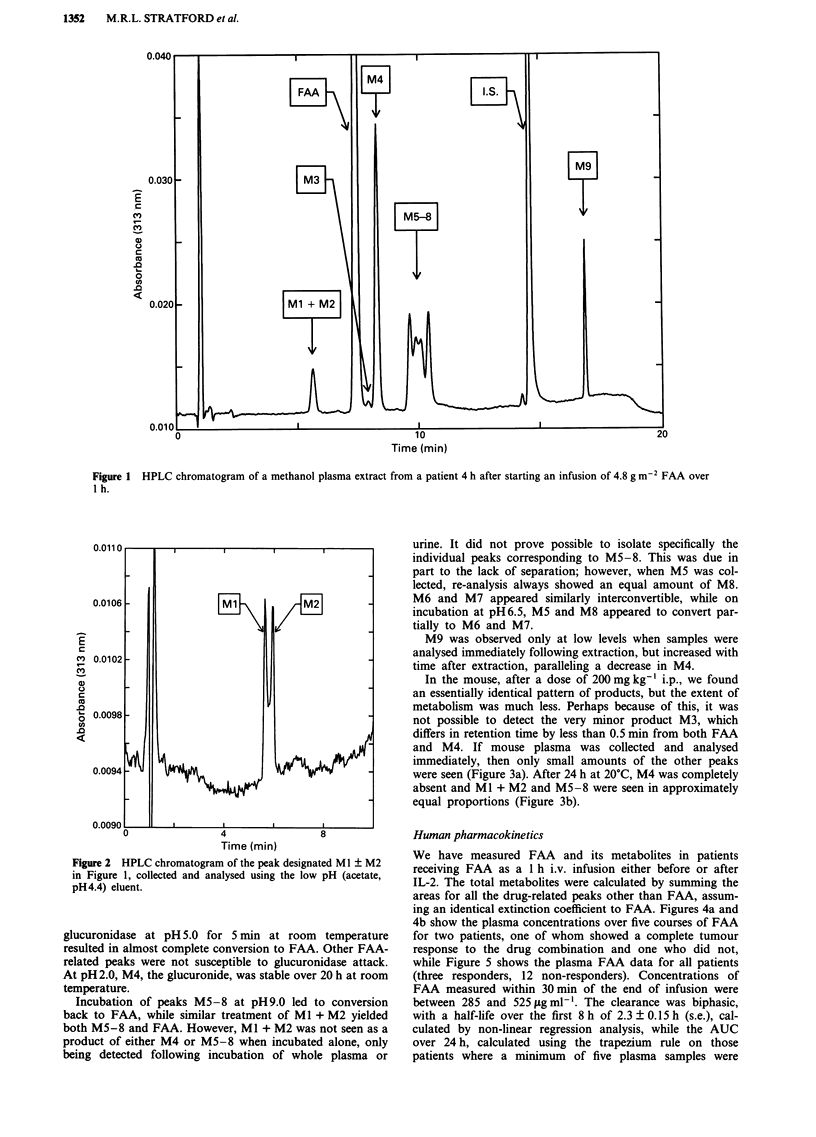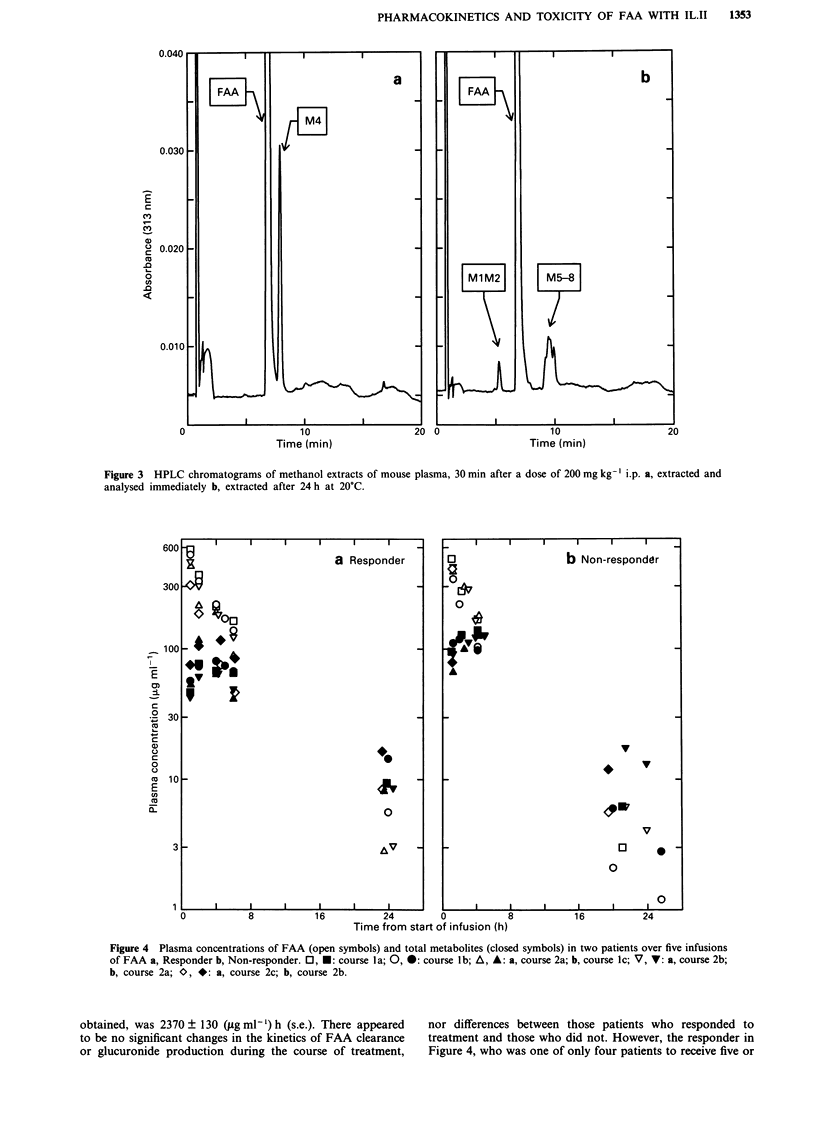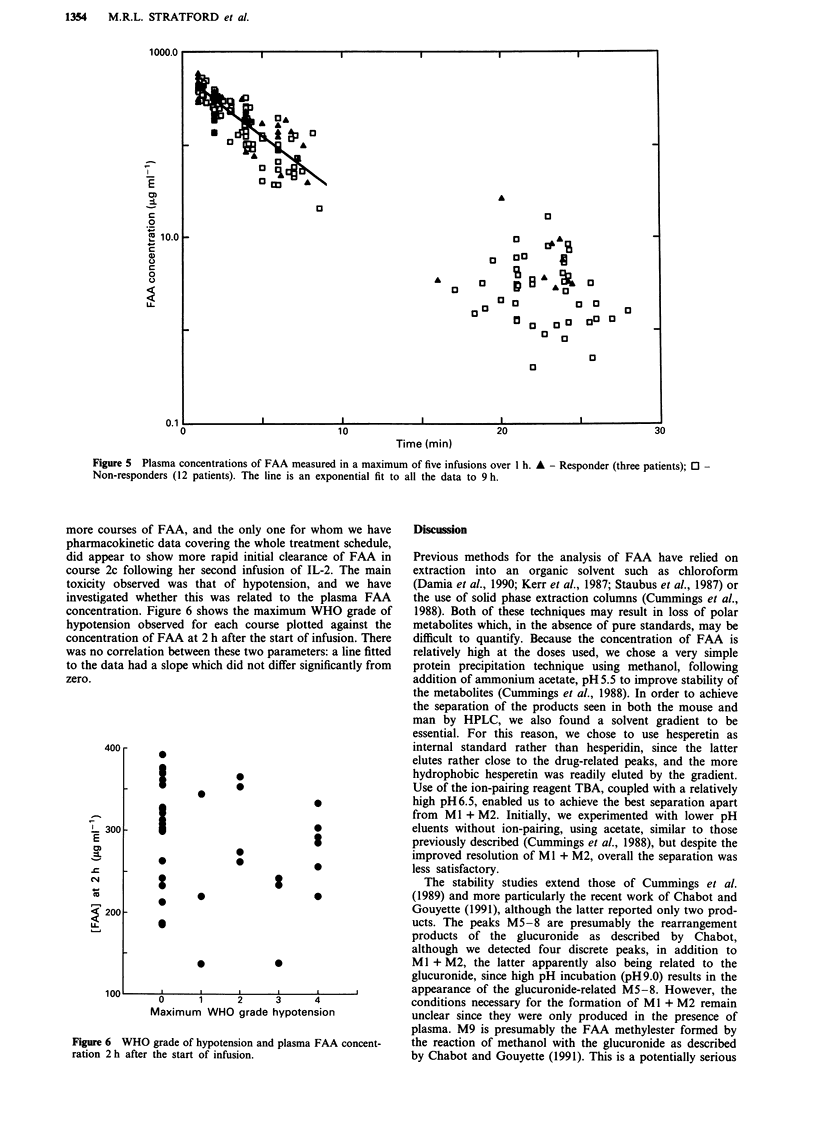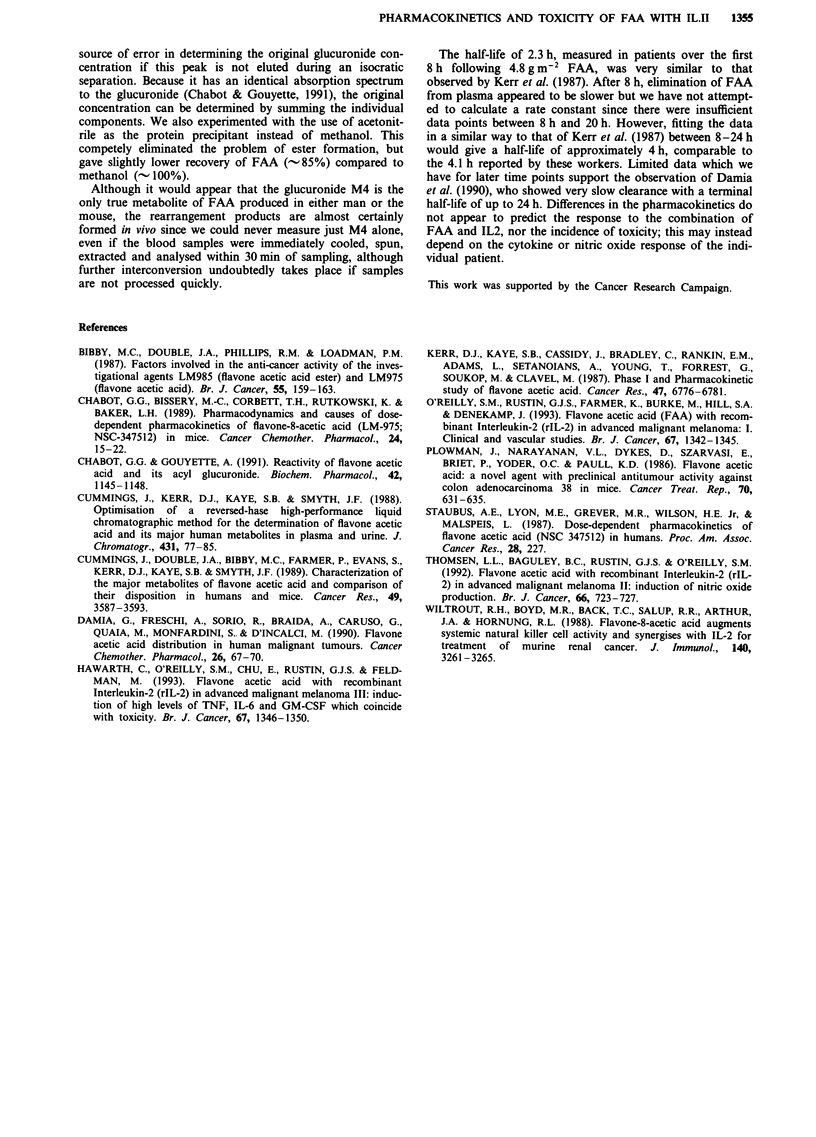# Flavone acetic acid (FAA) with recombinant interleukin-2 (rIL-2) in advanced malignant melanoma. IV: Pharmacokinetics and toxicity of flavone acetic acid and its metabolites.

**DOI:** 10.1038/bjc.1993.250

**Published:** 1993-06

**Authors:** M. R. Stratford, G. J. Rustin, M. F. Dennis, R. R. Watfa, N. Howells, S. M. O'Reilly

**Affiliations:** Cancer Research Campaign Gray Laboratory, Northwood, Middlesex.

## Abstract

Flavone acetic acid (FAA) was administered at a dose of 4.8 g m-2 over 1 h to patients with advanced malignant disease in combination with Interleukin II. A new high performance liquid chromatography method is described to determine both the parent compound and eight drug-related products, and the conditions required to determine these components in plasma are discussed. The half-life over the first 8 h was 2.3 h, but the terminal clearance of the drug was extremely slow. Severe (WHO Grade 4) hypotension was observed in some patients. However, incidence of this did not appear to be associated with any differences in FAA plasma concentrations, nor were there differences in FAA clearance between those patients whose tumour responded to the drug combination and those who did not.


Br. J. Cancer (1993), 67, 1351-1355 © Macmillan Press Ltd., 1993

### Flavone acetic acid (FAA) with recombinant interleukin-2 (rIL-2) in advanced malignant melanoma IV: Pharmacokinetics and toxicity of flavone acetic acid and its metabolites

M.R.L. Stratford[1], G.J.S. Rustin[2,3], M.F. Dennis[1], R.R. Watfa[1], N. Howells[2] & S.M. O'Reilly[3]

[1]*Cancer Research Campaign Gray Laboratory and* [2]*Regional Cancer Centre, Mount Vernon Hospital, Northwood, Middlesex HA6 2JR;* [3]*Cancer Research Campaign Laboratories, Department of Medical Oncology, Charing Cross Hospital, Fulham Palace Road, London W2 8RF.*

**Summary** Flavone acetic acid (FAA) was administered at a dose of 4.8 g m$^{-2}$ over 1 h to patients with advanced malignant disease in combination with Interleukin II. A new high performance liquid chromatography method is described to determine both the parent compound and eight drug-related products, and the conditions required to determine these components in plasma are discussed. The half-life over the first 8 h was 2.3 h, but the terminal clearance of the drug was extremely slow. Severe (WHO Grade 4) hypotension was observed in some patients. However, incidence of this did not appear to be associated with any differences in FAA plasma concentrations, nor were there differences in FAA clearance between those patients whose tumour responded to the drug combination and those who did not.

Flavone acetic acid (FAA) is a flavonoid which has been found to have high activity against solid murine tumours (Plowman *et al.*, 1986), although it is inactive against leukaemias and is not directly cytotoxic *in vitro* (Bibby *et al.*, 1987). In a murine renal carcinoma model, it has been shown to have a synergistic antitumour effect when combined with Interleukin 2(rIL-2) (Wiltrout *et al.*, 1988), which may indicate an involvement of immune effector cells. However, it is possible that metabolism of FAA is required for its action, and Chabot *et al.* (1989) have reported the occurrence of cytotoxic metabolites of FAA. A phase I trial of weekly 1 h infusions of FAA combined with 5 day infusions of recombinant human rIL-2 has been performed, and this paper describes the effect of rIL-2 on FAA metabolism and investigates whether the toxicity observed correlates with any changes in FAA pharmacokinetics or metabolism. A new HPLC method for the measurement of FAA and its metabolites is also presented, since published methods (Bibby *et al.*, 1987; Chabot *et al.*, 1989; Chabot & Gouyette, 1991; Cummings *et al.*, 1988; Damia *et al.*, 1990; Kerr *et al.*, 1987) do not resolve adequately all the peaks which we have observed. This technique enables us to measure FAA, its glucuronide, seven degradation products, and a minor product which we have been unable to characterise further. The clinical data, histology of post treatment tumour biopsy samples, nitrate levels and cytokine responses will be presented separately.

### Materials and methods

All chemicals were from BDH Ltd. except for methanol, acetonitrile and tetrahydrofuran (Rathburn Chemicals Ltd.), hesperetin (Sigma) and tetrabutyl ammonium hydroxide (TBA) (Fisons Ltd.).

Fifteen patients with metastatic melanoma were entered into the trial of FAA (Lipha Lyonnaise Industrielle, Lyons, France), combined with rIL-2 (Proleukin, Eurocetus, Amsterdam, Netherlands). Prior to treatment, all patients had progressive disease and a performance status $\leqslant 3$ on a 5-grade scale according to WHO criteria. The treatment protocol, which had been approved by the local ethical committee on human experimentation, was modified during the trial to reduce the severity of side effects (i.e. hypotension). The first eight patients were given FAA (4.8 g m$^{-2}$) as a 1 h infusion in 500 ml 0.9% saline without urine alkalinisation on days 1, 8 and 15 (courses 1a, b, c). rIL-2 ($6-18 \times 10^6$ international units m$^{-2}$ day$^{-1}$) was given as a continuous infusion on days 8-12 and 15-19. For the next seven patients, FAA and IL-2 were given as described except that rIL-2 was given on days 8-12 only. Following treatment, patients were assessed for clinical responses according to standard WHO criteria and the treatment was repeated after 2 weeks (courses 2a, b, c) unless evidence of disease progression was observed.

Blood samples were collected into heparinised tubes and immediately cooled on ice prior to centrifugation at 4°C. The samples were then stored at −70°C, normally within 1 h of sampling. To avoid prolonged thawing times, in the latter half of the trial, plasma samples were aliquoted into tubes ready for extraction prior to freezing. For analysis, to 100 µl (200 µl, > 12 h post FAA) plasma was added 100 nmol hesperetin (1 mM in methanol), 1 ml methanol, and 50 µl 1M ammonium acetate pH 5.5, and the samples mixed after each addition. After centrifugation at 4°C, the sample was ready for analysis by HPLC using a Waters 840 data analysis system with a WISP autosampler, a Novapak C18 column (15 cm × 4 mm), and a 441 detector operating at 313 nm. Most of the separations were carried out using two solvents: A: 5 mM TBA, 20 mM sodium dihydrogen orthophosphate (pH 6.5); B: 60% acetonitrile, 10% tetrahydrofuran, 30% water, with linear gradients of 25-34% B, 0-9 min, 34-60% B, 9-15 min, 60-25% B, 15-16 min. The flow rate was 1.5 ml min$^{-1}$ and the run time 20 min. The low pH eluent was: A: 5 mM sodium acetate, 0.5 ml l$^{-1}$ glacial acetic acid (pH 4.4); B: 40% acetonitrile, 10% tetrahydrofuran, 50% methanol, with a linear gradent of 15%-30% in 6 min (Hawarth, 1993; O'Reilly, 1993; Thomsen, 1992).

### Results

### *Chromatography*

A chromatogram of a human plasma sample taken 4 h after the start of infusion of FAA is shown in Figure 1, and illustrates the large number of drug-related peaks resolved. The first peak is labelled M1 + M2 since it does in fact comprise two components. This is shown in Figure 2 where the peak has been collected and injected into the low pH eluent, which resolves it into two peaks. The stability of the main metabolite M4 was also studied under various conditions following collection of the peak. At pH 6.5 (th pH of the eluent), after 20 h at room temperature, ~40% of the peak was lost, with the majority going to M5-8 and a small amount converted back to FAA. Incubation with β-

Correspondence: M.R.L. Stratford.
Received 25 February 1992; and in revised form 21 May 1992.

**Figure 1** HPLC chromatogram of a methanol plasma extract from a patient 4 h after starting an infusion of 4.8 g $m^{-2}$ FAA over 1 h.

**Figure 2** HPLC chromatogram of the peak designated M1 ± M2 in Figure 1, collected and analysed using the low pH (acetate, pH4.4) eluent.

glucuronidase at pH5.0 for 5 min at room temperature resulted in almost complete conversion to FAA. Other FAA-related peaks were not susceptible to glucuronidase attack. At pH2.0, M4, the glucuronide, was stable over 20 h at room temperature.

Incubation of peaks M5–8 at pH9.0 led to conversion back to FAA, while similar treatment of M1 + M2 yielded both M5–8 and FAA. However, M1 + M2 was not seen as a product of either M4 or M5–8 when incubated alone, only being detected following incubation of whole plasma or urine. It did not prove possible to isolate specifically the individual peaks corresponding to M5–8. This was due in part to the lack of separation; however, when M5 was collected, re-analysis always showed an equal amount of M8. M6 and M7 appeared similarly interconvertible, while on incubation at pH6.5, M5 and M8 appeared to convert partially to M6 and M7.

M9 was observed only at low levels when samples were analysed immediately following extraction, but increased with time after extraction, paralleling a decrease in M4.

In the mouse, after a dose of 200 mg $kg^{-1}$ i.p., we found an essentially identical pattern of products, but the extent of metabolism was much less. Perhaps because of this, it was not possible to detect the very minor product M3, which differs in retention time by less than 0.5 min from both FAA and M4. If mouse plasma was collected and analysed immediately, then only small amounts of the other peaks were seen (Figure 3a). After 24 h at 20°C, M4 was completely absent and M1 + M2 and M5–8 were seen in approximately equal proportions (Figure 3b).

*Human pharmacokinetics*

We have measured FAA and its metabolites in patients receiving FAA as a 1 h i.v. infusion either before or after IL-2. The total metabolites were calculated by summing the areas for all the drug-related peaks other than FAA, assuming an identical extinction coefficient to FAA. Figures 4a and 4b show the plasma concentrations over five courses of FAA for two patients, one of whom showed a complete tumour response to the drug combination and one who did not, while Figure 5 shows the plasma FAA data for all patients (three responders, 12 non-responders). Concentrations of FAA measured within 30 min of the end of infusion were between 285 and 525 μg $ml^{-1}$. The clearance was biphasic, with a half-life over the first 8 h of 2.3 ± 0.15 h (s.e.), calculated by non-linear regression analysis, while the AUC over 24 h, calculated using the trapezium rule on those patients where a minimum of five plasma samples were

Figure 3 HPLC chromatograms of methanol extracts of mouse plasma, 30 min after a dose of 200 mg kg$^{-1}$ i.p. a, extracted and analysed immediately b, extracted after 24 h at 20°C.

Figure 4 Plasma concentrations of FAA (open symbols) and total metabolites (closed symbols) in two patients over five infusions of FAA a, Responder b, Non-responder. □, ■: course 1a; ○, ●: course 1b; △, ▲: a, course 2a; b, course 1c; ▽, ▼: a, course 2b; b, course 2a; ◇, ◆: a, course 2c; b, course 2b.

obtained, was 2370 ± 130 (µg ml$^{-1}$) h (s.e.). There appeared to be no significant changes in the kinetics of FAA clearance or glucuronide production during the course of treatment, nor differences between those patients who responded to treatment and those who did not. However, the responder in Figure 4, who was one of only four patients to receive five or

**Figure 5** Plasma concentrations of FAA measured in a maximum of five infusions over 1 h. ▲ – Responder (three patients); □ – Non-responders (12 patients). The line is an exponential fit to all the data to 9 h.

more courses of FAA, and the only one for whom we have pharmacokinetic data covering the whole treatment schedule, did appear to show more rapid initial clearance of FAA in course 2c following her second infusion of IL-2. The main toxicity observed was that of hypotension, and we have investigated whether this was related to the plasma FAA concentration. Figure 6 shows the maximum WHO grade of hypotension observed for each course plotted against the concentration of FAA at 2 h after the start of infusion. There was no correlation between these two parameters: a line fitted to the data had a slope which did not differ significantly from zero.

**Figure 6** WHO grade of hypotension and plasma FAA concentration 2 h after the start of infusion.

### Discussion

Previous methods for the analysis of FAA have relied on extraction into an organic solvent such as chloroform (Damia *et al.*, 1990; Kerr *et al.*, 1987; Staubus *et al.*, 1987) or the use of solid phase extraction columns (Cummings *et al.*, 1988). Both of these techniques may result in loss of polar metabolites which, in the absence of pure standards, may be difficult to quantify. Because the concentration of FAA is relatively high at the doses used, we chose a very simple protein precipitation technique using methanol, following addition of ammonium acetate, pH 5.5 to improve stability of the metabolites (Cummings *et al.*, 1988). In order to achieve the separation of the products seen in both the mouse and man by HPLC, we also found a solvent gradient to be essential. For this reason, we chose to use hesperetin as internal standard rather than hesperidin, since the latter elutes rather close to the drug-related peaks, and the more hydrophobic hesperetin was readily eluted by the gradient. Use of the ion-pairing reagent TBA, coupled with a relatively high pH 6.5, enabled us to achieve the best separation apart from M1 + M2. Initially, we experimented with lower pH eluents without ion-pairing, using acetate, similar to those previously described (Cummings *et al.*, 1988), but despite the improved resolution of M1 + M2, overall the separation was less satisfactory.

The stability studies extend those of Cummings *et al.* (1989) and more particularly the recent work of Chabot and Gouyette (1991), although the latter reported only two products. The peaks M5–8 are presumably the rearrangement products of the glucuronide as described by Chabot, although we detected four discrete peaks, in addition to M1 + M2, the latter apparently also being related to the glucuronide, since high pH incubation (pH 9.0) results in the appearance of the glucuronide-related M5–8. However, the conditions necessary for the formation of M1 + M2 remain unclear since they were only produced in the presence of plasma. M9 is presumably the FAA methylester formed by the reaction of methanol with the glucuronide as described by Chabot and Gouyette (1991). This is a potentially serious source of error in determining the original glucuronide concentration if this peak is not eluted during an isocratic separation. Because it has an identical absorption spectrum to the glucuronide (Chabot & Gouyette, 1991), the original concentration can be determined by summing the individual components. We also experimented with the use of acetonitrile as the protein precipitant instead of methanol. This competely eliminated the problem of ester formation, but gave slightly lower recovery of FAA (~85%) compared to methanol (~100%).

Although it would appear that the glucuronide M4 is the only true metabolite of FAA produced in either man or the mouse, the rearrangement products are almost certainly formed *in vivo* since we could never measure just M4 alone, even if the blood samples were immediately cooled, spun, extracted and analysed within 30 min of sampling, although further interconversion undoubtedly takes place if samples are not processed quickly.

The half-life of 2.3 h, measured in patients over the first 8 h following 4.8 g $m^{-2}$ FAA, was very similar to that observed by Kerr *et al.* (1987). After 8 h, elimination of FAA from plasma appeared to be slower but we have not attempted to calculate a rate constant since there were insufficient data points between 8 h and 20 h. However, fitting the data in a similar way to that of Kerr *et al.* (1987) between 8–24 h would give a half-life of approximately 4 h, comparable to the 4.1 h reported by these workers. Limited data which we have for later time points support the observation of Damia *et al.* (1990), who showed very slow clearance with a terminal half-life of up to 24 h. Differences in the pharmacokinetics do not appear to predict the response to the combination of FAA and IL2, nor the incidence of toxicity; this may instead depend on the cytokine or nitric oxide response of the individual patient.

This work was supported by the Cancer Research Campaign.

### References

BIBBY, M.C., DOUBLE, J.A., PHILLIPS, R.M. & LOADMAN, P.M. (1987). Factors involved in the anti-cancer activity of the investigational agents LM985 (flavone acetic acid ester) and LM975 (flavone acetic acid). *Br. J. Cancer*, **55**, 159–163.

CHABOT, G.G., BISSERY, M.-C., CORBETT, T.H., RUTKOWSKI, K. & BAKER, L.H. (1989). Pharmacodynamics and causes of dose-dependent pharmacokinetics of flavone-8-acetic acid (LM-975; NSC-347512) in mice. *Cancer Chemother. Pharmacol.*, **24**, 15–22.

CHABOT, G.G. & GOUYETTE, A. (1991). Reactivity of flavone acetic acid and its acyl glucuronide. *Biochem. Pharmacol.*, **42**, 1145–1148.

CUMMINGS, J., KERR, D.J., KAYE, S.B. & SMYTH, J.F. (1988). Optimisation of a reversed-hase high-performance liquid chromatographic method for the determination of flavone acetic acid and its major human metabolites in plasma and urine. *J. Chromatogr.*, **431**, 77–85.

CUMMINGS, J., DOUBLE, J.A., BIBBY, M.C., FARMER, P., EVANS, S., KERR, D.J., KAYE, S.B. & SMYTH, J.F. (1989). Characterization of the major metabolites of flavone acetic acid and comparison of their disposition in humans and mice. *Cancer Res.*, **49**, 3587–3593.

DAMIA, G., FRESCHI, A., SORIO, R., BRAIDA, A., CARUSO, G., QUAIA, M., MONFARDINI, S. & D'INCALCI, M. (1990). Flavone acetic acid distribution in human malignant tumours. *Cancer Chemother. Pharmacol.*, **26**, 67–70.

HAWARTH, C., O'REILLY, S.M., CHU, E., RUSTIN, G.J.S. & FELDMAN, M. (1993). Flavone acetic acid with recombinant Interleukin-2 (rIL-2) in advanced malignant melanoma III: induction of high levels of TNF, IL-6 and GM-CSF which coincide with toxicity. *Br. J. Cancer*, **67**, 1346–1350.

KERR, D.J., KAYE, S.B., CASSIDY, J., BRADLEY, C., RANKIN, E.M., ADAMS, L., SETANOIANS, A., YOUNG, T., FORREST, G., SOUKOP, M. & CLAVEL, M. (1987). Phase I and Pharmacokinetic study of flavone acetic acid. *Cancer Res.*, **47**, 6776–6781.

O'REILLY, S.M., RUSTIN, G.J.S., FARMER, K., BURKE, M., HILL, S.A. & DENEKAMP, J. (1993). Flavone acetic acid (FAA) with recombinant Interleukin-2 (rIL-2) in advanced malignant melanoma: I. Clinical and vascular studies. *Br. J. Cancer*, **67**, 1342–1345.

PLOWMAN, J., NARAYANAN, V.L., DYKES, D., SZARVASI, E., BRIET, P., YODER, O.C. & PAULL, K.D. (1986). Flavone acetic acid: a novel agent with preclinical antitumour activity against colon adenocarcinoma 38 in mice. *Cancer Treat. Rep.*, **70**, 631–635.

STAUBUS, A.E., LYON, M.E., GREVER, M.R., WILSON, H.E. Jr, & MALSPEIS, L. (1987). Dose-dependent pharmacokinetics of flavone acetic acid (NSC 347512) in humans. *Proc. Am. Assoc. Cancer Res.*, **28**, 227.

THOMSEN, L.L., BAGULEY, B.C., RUSTIN, G.J.S. & O'REILLY, S.M. (1992). Flavone acetic acid with recombinant Interleukin-2 (rIL-2) in advanced malignant melanoma II: induction of nitric oxide production. *Br. J. Cancer*, **66**, 723–727.

WILTROUT, R.H., BOYD, M.R., BACK, T.C., SALUP, R.R., ARTHUR, J.A. & HORNUNG, R.L. (1988). Flavone-8-acetic acid augments systemic natural killer cell activity and synergises with IL-2 for treatment of murine renal cancer. *J. Immunol.*, **140**, 3261–3265.